# Development and Application of Medicine-Engineering Integration in the Rehabilitation of Traumatic Brain Injury

**DOI:** 10.1155/2021/9962905

**Published:** 2021-06-12

**Authors:** Qingyong Wang, Weibo Sun, Yuanyuan Qu, Chuwen Feng, Delong Wang, Hongna Yin, Chaoran Li, Zhongren Sun, Dongwei Sun

**Affiliations:** ^1^Heilongjiang University of Chinese Medicine, 24 Heping Road, Harbin, China; ^2^Harbin Medical University, 157 Baojian Road, Harbin, China; ^3^First Affiliated Hospital, Heilongjiang University of Chinese Medicine, 26 Heping Road, Harbin, China; ^4^Second Affiliated Hospital, Heilongjiang University of Chinese Medicine, 411 Guogeli Road, Harbin, China 150040; ^5^Department of Rehabilitation, Fifth Affiliated Hospital of Shenzhen University, Shenzhen, China 510090

## Abstract

The rapid progress of the combination of medicine and engineering provides better chances for the clinical treatment and healthcare engineering. Traumatic brain injury (TBI) and its related symptoms have become a major global health problem. At present, these techniques has been widely used in the rehabilitation of TBI. In this review article, we summarizes the progress of the combination of medicine and industry in the rehabilitation of traumatic brain injury in recent years, mainly from the following aspects: artificial intelligence (AI), brain-computer interfaces (BCI), noninvasive brain stimulation (NIBS), and wearable-assisted devices. We believe the summary of this article can improve insight into the combination of medicine and industry in the rehabilitation of traumatic brain injury.

## 1. Introduction

**Figure 1:**
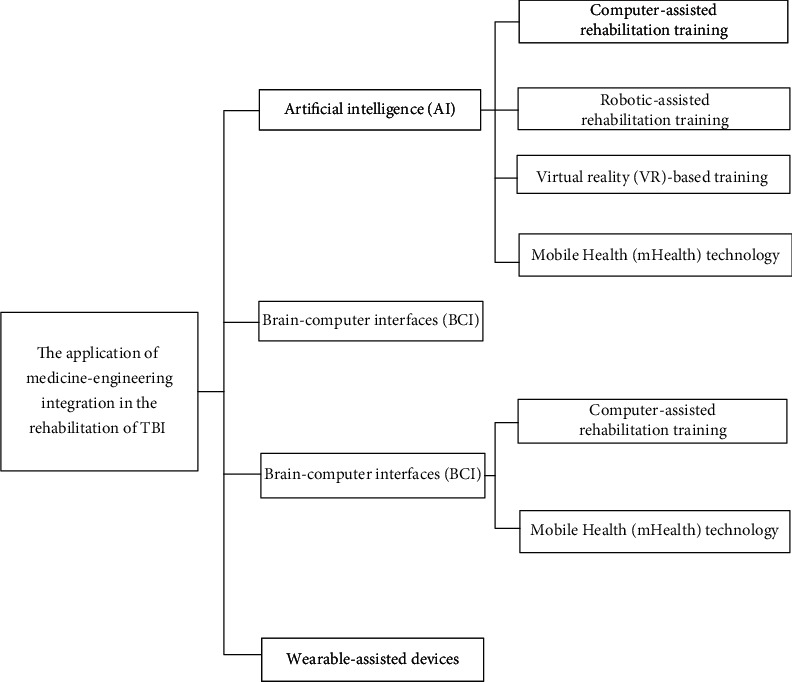
The application of Medicine-Engineering integration in the rehabilitation of TBI.

With the development of the society, traumatic brain injury (TBI) is gaining more attention because of its higher rates of morbidity and mortality. It is defined as a traumatic structural or physiological disruption of brain function, mainly caused by an external physical force. Traumatic brain injury (TBI) is the mainly common cause of death and disability in those aged under 40 years in the UK [1]. TBI still plague millions of peoples around the world every year [2]. Though the efforts for exploring therapeutic strategies for the rehabilitation of TBI have been taken over the past few decades, there is still a lack of effective treatment for it, and the treatment of TBI is far from satisfactory. Meanwhile, the combination of medicine and engineering brings new rehabilitation methods to TBI patients. The combination of medicine and engineering is a newly developed interdisciplinary subject in recent years, which is the product of the integration and innovation of medical science and engineering science, and it brings new idea to this significant public health issue. In this review article, we outline recent breakthroughs in the combination of medicine and engineering and their applications in the rehabilitation of TBI. We believe the summary of this article can improve insight into the usage of the combination of medicine and engineering in TBI's rehabilitation. As shown in Figure 1, we mainly describe advances including artificial intelligence (AI), brain-computer interfaces, and noninvasive brain stimulation (NIBS) et al.

## 2. Artificial Intelligence (AI)

Artificial intelligence is a broad interdisciplinary field, which is composed of logic, cognitive psychology, decision theory, neuroscience, linguistics, computer engineering, and so on [3]. As time goes by, artificial intelligence is gradually changing the clinical practice of medicine. With the continuous development of digital data acquisition, machine learning, and computing foundation, the application of artificial intelligence is expanding to the field previously considered as human experts [4]. The fields of traumatic brain injury (TBI) and artificial intelligence (AI) have been studied for a long time. In recent years, artificial intelligence (AI) is applied in the rehabilitation of traumatic brain injury (TBI) widely. The application of AI in TBI's rehabilitation mainly including computer-assisted rehabilitation training, robotic-assisted rehabilitation training, virtual reality (VR), and smart mobile technology.

### 2.1. Computer-Assisted Rehabilitation Training

Cognitive dysfunction is one of the main consequences of traumatic brain injury (TBI). Cognitive impairment leads to a serious decline in the quality of life after TBI and is involved in the impact of depressive symptoms on emotional role functioning [5]. By far, computer-assisted rehabilitation training has been widespread application in the cognitive rehabilitation in people with traumatic brain injury (TBI).

A study of 35 patients with traumatic or vascular brain injury found that compared with conventional treatment, cognitive training can effectively improve the rehabilitation effect after TBI, which brings new hope for TBI patients [6]. In this study, they detail an introduction to how computer-assisted cognitive rehabilitation (CACR) uses multimedia and informatics resources directly to utilize specific hardware systems and software to activate the expression of impaired neurocognitive function through specific programs. In 2013, Cruz et al. developed a new Web-based rehabilitation tool called “COGWEB” that intensive cognitive training is provided at home at an affordable cost under clinical prescription and supervision, compared with previous traditional cognitive rehabilitation technique. The COGWEB system motivated the treatment positive of patients with traumatic brain injury [7]. Dou et al. conducted a clinical study to evaluate the efficacy of computerized error-free learning memory rehabilitation program for Chinese patients with traumatic brain injury (TBI) in 2006. The result indicated that computer-assisted memory rehabilitation (CAMG) improved the memories of patients with TBI [8]. Another study examined the effectiveness of computer-assisted cognitive rehabilitation (CACR) in patients with traumatic brain injury (TBI), the result found that CACR significantly improved the cognition of TBI patients, but the extent and nature of these gains remains to be further studied [9]. In the above, we can know that computer-based interventions seem to hold great promise in improving working memory in people with acquired traumatic brain injury, but it is more commonly used for cognitive rehabilitation after traumatic brain injury and the durability of its efficacy remains to be studied. Meanwhile, a systematic review and meta-analysis found that computer-assisted rehabilitation training might be a benefit to improve visual and verbal working memory for TBI patients, but other domains such as attention, processing speed, and executive functions were not benefited by the interventions [10]. We can learn that the application of computer-assisted cognitive rehabilitation (CACR) in TBI rehabilitation remains to be further explored. The advantages and disadvantages of computer-assisted rehabilitation training are summarized in Table 1.

### 2.2. Robotic-Assisted Rehabilitation Training

In the past decades, rehabilitation robots have been rapid and vast developments. As a relatively young and rapidly developing field, rehabilitation robots are increasingly infiltrating into the clinical environment [11]. An increasing number of patients are suffering from limb motor dysfunction, which may be caused by stroke-related nerve damage, traumatic brain injury, or multiple sclerosis [12]. The robotic-assisted rehabilitation therapy can deliver high-quality training to enhance the recovery process and promote the recovery of limb function.

One study presented the application of novel self-feeding robots in TBI rehabilitation, and the self-feeding robots improved the activities of daily living of patients with TBI [13]. In another study [14], Zeng et al. recruited 2 patients with TBI to evaluate the application of collaborative wheelchair assistant system (CWAS) in cerebral palsy and traumatic brain injury users. The results showed that CWAS improved the patients' motor function. Meanwhile, robotic gait training is applied in gait impairment after TBI widely. In 2016, Stam et al. [15] reported a case study that uses robotic gait assistive technology is beneficial for the rehabilitation of participants with TBI. Another study also confirmed that robotic-assisted locomotor training can improve the locomotor performance of patients with TBI [16]. In a study of 16 participants with TBI receiving 18 robotic gait training duration six weeks, the result of this study demonstrated that robotic-assisted treadmill training (RATT) improves step length greater than manually assisted treadmill training (MATT) [17]. Ozgur et al. designed Configurable Arm Rehabilitation Games for patients with chronic upper limb impairment after TBI. The gamified rehabilitation platform using tangible robots can well promote the rehabilitation of limb function [18]. In addition, Brewer et al. designed a model called visual feedback distortion for the rehabilitation of patients with TBI [19]. Two patients were enrolled in a six-week rehabilitation program. In this case, patients who are physically capable of advancing in rehabilitation did not prevent them because they had reached habitual or self-imposed limits, and each patient followed the level of visual feedback distortion higher than her performance predicted in the initial evaluation.

In the above, we can learn that robotic-assisted rehabilitation training pays more attention to the limb motor dysfunction after TBI; in my opinion, we should focus on the robot research of hand fine motion rehabilitation in the future. The advantages and disadvantages of robotic-assisted rehabilitation training are summarized in Table 2.

### 2.3. Virtual Reality- (VR-) Based Training

Virtual reality (VR) is a new and developing technology, which combines the characteristics of VR technology such as autonomy, interactivity, and existence with rehabilitation training. VR is described as “an advanced human-computer interaction mode that allows users to interact in a natural way with a computer-based environment for training and full immersion.” [20]. VR is now offering more new treatment measures for patients with TBI.

A study of 33 TBI patients examined the usability of a virtual reality driving simulator [21]. All patients were asked to perform a VR driver rehabilitation (VR-DR) system and completed the related User Feedback Questionnaire. The result found that the VR-DR system could be well applied to the rehabilitation training of TBI patients. Another study tested the availability and efficacy of a newly developed virtual reality- (VR-) based community living skills training program for people with TBI, and the result suggested the produced positive changes in TBI subjects [22]. The study of 18 patients with severe TBI found that through two consecutive days of 3D-cancellation in an interactive virtual environment, VR and robotics technology improved the attention impairment in patients with TBI [23]. Similarly, Bisson's study also shown that rehabilitation training in an interactive visuo-haptic environment may be beneficial to the early recovery of attention in patients with TBI [24]. Additionally, virtual reality (VR) has been used in conjunction with robotics, biofeedback training, and modern multitouch technology. In 2019, Maggio et al. [25] conducted a study of 56 participants with TBI; the experimental group underwent rehabilitation training with Lokomat Pro, equipped with a VR screen; the rehabilitation protocol consisted of a total of 40 training sessions. Ultimately, the result supported that Lokomat plus virtual reality can improve the cognitive and behavioral functions in participants with TBI. Using multitouch-multiuser tabletop (MMT) devices: Snowflake MultiTeach (MT) and Diamond Touch Table (DTT), coupled with MediqVR virtual reality (VR) platform, and computer-based interactive applications were applied in rehabilitation design; the research found that MediqVR training improves the patients' intuition, communication and expression ability, enable them to carry out social activities naturally, and reduce the patients' social anxiety [26]. We summarized the advantages and disadvantages of virtual reality- (VR-) based training in Table 3.

### 2.4. Mobile Health (mHealth) Technology

TBI can lead to severe motor, cognitive, and emotional disturbance, and the rehabilitation of TBI is a long process. Fortunately, Mobile Health (mHealth) is an emerging technology, which can help to diagnose and manage patients with TBI greater.

One study explored that the effectiveness of a prospective memory assists method combining smartphones with Web-based calendars in community-dwelling patients with traumatic brain injury [27]. Patients were asked to use a windows phone- (version 7.5) based smartphone as the primary memory compensation strategy during the intervention phase, avoiding the use of existing assistive tools and strategies. A study of 13 patients received a group-based therapy for six weeks, and the result supported that the smartphone improved patients' memory problems significantly. Another study assessed compliance with an 8-week intervention daily ecological momentary assessments (EMA) conducted via a smartphone application, and the result also demonstrated the efficacy of mHealth system [28]. There are also reports of new technologies that Interactive iBook-Based Patient Education be applied to improve the self-reported measures of patient and family knowledge, and it is helpful to improve the potential anxiety and other symptoms after TBI [29]. The advantages and disadvantages of Mobile Health (mHealth) Technology are summarized in Table 4.

## 3. Brain-Computer Interfaces (BCI)

Brain-computer interfaces (BCI) are developing into a possible method to replace the brain's normal output pathways of peripheral nerves and muscles, and allowing paralytic patients can use a new method of communication and computer control, which has developed significantly over the past several decades [30]. Brain-computer interface (BCI) can convert brain signals obtained by noninvasive and invasive methods into control signals of some external devices, such as computer cursor or robot limb [31].

Many clinical studies confirmed the effectiveness of BCI in the rehabilitation of patients with TBI [32, 33]. Morrison et al. applied Hopfield neuronal networks to prevent the loss of memory in TBI patients by using cerebral organoids or external microelectronics, which provide a starting point for new treatment strategies [34]. In addition, the integration of brain-computer interface (BCI) and functional electrical stimulation (FES) technologies also bring better treatment [35]. Although the BCI technology has advanced significantly over the years, it still faces many challenges. These challenges mainly include signal degradation (from implanted recording electrodes), accuracy and robustness of neural decoding algorithms over time, miniaturization of the system, adverse events from using FES, and ease of use of whole system et al., so the application of BCI in TBI rehabilitation still need further exploration. We summarized the advantages and disadvantages of brain-computer interfaces (BCI) in Table 5.

## 4. Noninvasive Brain Stimulation (NIBS)

In the past few years, the extensive use of noninvasive brain stimulation (NIBS) technology has led to a significant development in our understanding of brain behavioral relationships [36]. As a potential treatment for neurological and psychiatric diseases, including traumatic brain injury, it has also received extensive attention [37]. NIBS mainly includes transcranial direct current stimulation (tDCS) and transcranial magnetic stimulation (TMS).

### 4.1. Transcranial Direct Current Stimulation (tDCS)

Transcranial direct current stimulation (tDCS) is one method of NIBS. It can increase or decrease cortical excitability according to different polarities (anode or cathode), regulate synaptic plasticity through long-term inhibition or enhancement, and promote long-term functional recovery [38]. It can provide a safe and noninvasive method for regulating neural excitability during neurorehabilitation.

In 2014, Middleton et al. [39] conduct a study, which enrolled 5 patients with chronic neurologic insult, which stroke or traumatic brain injury more than 6 months. Participants were requested to complete 24 courses (40 minutes, three times a week) of upper limb physical therapy (UE-PT) and to perform bihemispheric tDCS on the motor cortex at a speed of 1.5 MA in the first 15 minutes of each course. The result indicated that this therapy improves patients' indicators significantly. The emergence of posttraumatic disorders of consciousness (DOC) increases the mortality of patients and restricts their rehabilitation. A double-blind RCT study has found that tDCS can effectively improve the consciousness disorder of the people with TBI [40]. The systematic review of transcranial direct current stimulation (tDCS) effects on the rehabilitation of traumatic brain injury (TBI) also supported that although tDCS has been used in clinical treatment, it still needs further improvement, and the after-effects of tDCS are mostly short lived [41]. The advantages and disadvantages of transcranial direct current stimulation (tDCS) are summarized in Table 6.

### 4.2. Repeated Transcranial Magnetic Stimulation (rTMS)

rTMS is another noninvasion method to stimulate the human brain. It can influence brain plasticity and cortical reorganization through stimulation-induced changes in neuronal excitability, and the treatment effects of rTMS on cortical excitability depend on the stimulation parameters applied, including the stimulus intensity, frequency, and duration of stimulation [42]. The low-frequency rTMS (<1 Hz) applied at the motor threshold or slightly suprathreshold intensities result in a suppression of cortical excitability, and high-frequency (≥5 Hz) suprathreshold stimulation will lead to increased cortical excitability [43, 44].

Disorders in memory and neural behavior are a common sequence of TBI. A study shows that low-field magnetic stimulation (LFMS) improved the cognitive and motor function of TBI mice significantly. The neuroprotective effect of LFMS may be achieved through the regulation of cellular prion protein (PrPc) and/or circadian rhythm-related proteins [45]. A case report found that rTMS could improve the neural activity, to regulate the neural activity, and/or to facilitate recovery in patients with disturbance of consciousness after TBI [46]. Another study confirmed that high-frequency transcranial magnetic stimulation could reduce the pain scores of patients with TBI and improve quality of life [47]. Headache is another common symptom after TBI; Leung et al. conducted a study to test the effectiveness of rTMS in headache after TBI; the result indicated that rTMS can alleviate the headache symptom and provide a transient mood-enhancing benefit [48].

Although rTMS has been widely used in the rehabilitation of TBI, there are still a lot of areas that need to improve. There is evidence that rTMS may be led to adverse events such as seizure [49], so we should consider the safety when we use it. The advantages and disadvantages of Repeated Transcranial Magnetic Stimulation (rTMS) are summarized in Table 7.

## 5. Wearable-Assisted Devices

Wearable-assistive devices have been used in the rehabilitation of neurological disorder such as traumatic brain injury widely. According to the report [50], Mikołajczyk et al. carried out the research and design of the system for elbow rehabilitation which consists of a single-degree-of-freedom (SDOF) solution and a single-axis stepper motor with a controller. They designed an exoskeleton, a wearable, external structure which can support or even replace the muscle actuation in the patient, and the system promotes the rehabilitation of upper limb function after TBI. Portable electronic aids have also developed rapidly in recent years. In the study [51], we learned that portable electronic aids may improve the function of patients with TBI in the areas of learning, organization, and initiation, but its clinical application may be limited by its high price and low clinical confidence. The advantages and disadvantages of wearable-assistive devices are summarized in Table 8.

## 6. Discussion

In this paper, we summarized the application of interdisciplinary combination between medicine and engineering in the rehabilitation of traumatic brain injury. With the rapid development of science and technology, the interdisciplinary combination between medicine and engineering technology brings new hope to TBI patients. Artificial intelligence (AI), brain-computer interfaces (BCI), noninvasive brain stimulation (NIBS), and wearable-assistive devices have been widely used in the rehabilitation of patients with TBI; meanwhile, there are still some areas that need to improve.

First, as summarized above, we can see that the application of these technologies lacks high evidence from clinical trials, so we should conduct more clinical trials to prove their effectiveness in the future. When it comes to the application of virtual reality (VR), we must consider the limitations of it. The application of virtual reality technology in clinical practice is mainly limited by two factors: accessibility and the cost of virtual tools [20]. Additionally, Many interdisciplinary combinations between medicine and engineering technologies lack standardized treatment procedures; we should make efforts to develop the individualized, precise treatments in the future.

Secondly, clinician attitudes are important which can affect the use of any assistive technology in the training and supporting for the rehabilitation of TBI patients, so we should improve the awareness of clinician for the new rehabilitation facility. Moreover, according to report [52], traditional Chinese medicine therapy especially acupuncture and moxibustion therapy is a benefit to the rehabilitation of TBI patient; He et al. showed that early application of acupuncture gets better effects on restoration of arousal function of the brain in patients with TBI than functional electrical stimulation. So whether we can further the role of acupuncture therapy.

## Figures and Tables

**Table 1:** The advantages and disadvantages of computer-assisted rehabilitation training.

Computer-assisted rehabilitation training	
Advantages	(1) Overall function increase significantly [6]. (2) The simplicity of its use and comfort [7].

Disadvantages	(1) Lack of studies to determine the possible side effects of these interventions [7].

**Table 2:** The advantages and disadvantages of robotic-assisted rehabilitation training.

Robotic-assisted rehabilitation training	
Advantages	(1) The increase in mobility is beneficial to learning, communication, motivation, and social interaction [14]. (2) Better steerability [14]. (3) Higher intensity gait therapy [16].

Disadvantages	(1) Lack of personalized robot-assisted training (e.g., speed parameter) [17].

**Table 3:** The advantages and disadvantages of virtual reality- (VR-) based training.

Virtual reality- (VR-) based training	
Advantages	(1) More usable and cheaper tools [20, 21]. (2) Well-tolerated [24].

Disadvantages	(1) Accessibility and the cost of virtual tools [20]. (2) VR assessment protocols appear to be primarily implemented for mild TBI [20]. (3) Eye fatigue [24]

**Table 4:** The advantages and disadvantages of Mobile Health (mHealth) Technology.

Mobile Health (mHealth) technology	
Advantages	(1) Low-cost [27]. (2) Allowed the clinician to provide focused and personalized information [29].

Disadvantages	(1) These data represent a small sample, broader TBI population should be exercised with caution [28].

**Table 5:** The advantages and disadvantages of brain-computer interfaces (BCI).

Brain-computer interfaces (BCI)	
Advantages	(1) Invasive BCI: High accuracy [31] (2) Increase remote access to rehabilitation supporting transition into home [32]. (3) Allows for a better control of the system as well as greater effects on brain reorganizations [33]. (4) Implantable BCIs have provided neural recordings with increased spacial resolutions [35].

Disadvantages	(1) Limited ability to represent more than two signal output choices [30]. (2) The risks and expenses associated with the surgery [31]. (3) Signal degradation (from implanted recordings electrodes), accuracy and robustness of neural decoding algorithms over time, miniaturization of the system, muscle fatigue when using FES, and overall system ease-of-use [35].

**Table 6:** The advantages and disadvantages of transcranial direct current stimulation (tDCS).

Transcranial direct current stimulation (tDCS)	
Advantages	(1) Relative ease of use and good safety profile [37]. (2) A safe, noninvasive technique [39]. (3) Stimulation was well-tolerated [39]. (4) It is a painless, noninvasive, easily applied, and effective therapy [40].

Disadvantages	(1) There is an ongoing debate about the precise neurophysiological processes that are stimulated by these techniques [36]. (2) They can only directly affect activity in cortical regions [36]. (3) Did not focus on possible late-occurring side effects or side (4) Effects that might be caused by intensified use [36]. (5) Lack of large-sample clinical trials [40].

**Table 7:** The advantages and disadvantages of Repeated Transcranial Magnetic Stimulation.

Repeated Transcranial Magnetic Stimulation (rTMS)	
Advantages	(1) A noninvasive and painless method [42, 46]. (2) A safe, noninvasive, effective therapeutic intervention [47, 49]. (3) No significant side effects [48].

Disadvantages	(1) The most clinically effective rTMS parameters and the optimal site for stimulation in TBI are not currently known [42]. (2) The limited knowledge regarding the side effects of TMS [42]. (3) TBI patients who receive rTMS may experience a mild headache after the treatment session [42]. (4) Lead a risk of temporary mild hearing loss due to its substantial volume [42]. (5) The high intensity of stimulation is not well tolerated [48].

**Table 8:** The advantages and disadvantages of wearable-assistive devices.

Wearable-assistive devices	
Advantages	(1) Safety, proven efficacy [50]. (2) High cost and low clinician confidence [51].

Disadvantages	(1) Lack high-h-quality evidence to assess its effectiveness [50]. (2) The advantages and disadvantages of technology are unknown [51].

## Data Availability

This article is a review article and does not contain relevant data.
